# Teeth of the Ancients

**Published:** 1870-05

**Authors:** 


### TEETH OF THE ANCIENTS.

Harper's Monthly Magazine for April, contains in its "Editors' Scientific Record" an article entitled "Diseased Teeth Among the Ancients." The remarks therein contained prompted me to make the attempt promised you while in Cincinnati last summer, *i. e.* to write you an occasional article from Florence, inasmuch as I have had the pleasure of examining teeth that figured at many hash festivals more than two thousand years ago. These teeth are from the Tombs of Volterra. Among the few remaining Etruscan cities, to Volterra is awarded the palm of being the most interesting. In most cities partaking of the Etruscan there is such a mass of "jumbled in" Roman reminissences, that one is at a loss how to attempt to assort the entire in order to arrive at distinct perceptibility.

Etruria, as has been proven, was inhabited by a civilized and intelligent people long before Rome was founded. The " entire " of Etruria was a confederacy of twelve cities, with their adjacent districts. Their people are supposed to have begun their reign of power in Italy about 1,000 years B. C. Their navy was, at a very early period an arm of power on the Mediterranean. Their vessels visited the most extreme coasts of this sea, and their commerce was found over what was considered at that period, the entire world. Proficient in art, they were as dreaded in war. Industrious in the peaceful pursuits of husbandry, they were ever as eager for the trial of prowess with any enemy, be they either Gaul or Roman. In architecture, they in their special branches have never found their equals. For instance, in walls, for the purpose of defense in case of war, in battlements and towers, their heavy masonry still stands, the admiration and wonder of every "son of the trowel."

And among these twelve cities, as I before remarked, was that of Volterra. In the many wars that followed in after years with the Roman powers, Volterra seems to have been sheltered by some kind hand, in as much as the finer relics of the ancient arts are still found and exposed, beautiful, entire and perfect. The general demolition that was visited upon the Etrurian confederacy, seems to have either passed by or spent itself at this place.

The first thing that strikes the traveler in almost any country is the "cities of the dead." From grave yards have descended through long vistas of centuries many interesting facts and historical romances. The tombs of most ancient cities—the resting places of their dead, are as instructive as they are sad. The sides of the stones are always more or less garnished with bas reliefs, or scenes, giving the observer some sort of an idea how matters were carried on, say 1,000 years before Christ was born. Some of our fair ones in America regard the "latest Paris fashions" as something nearly divine, or should be made so. And in the study of that plait or the roll of that enormous knot, that the Paris fashion plate for 1870 teaches them is the latest, they little think that just 2870 years ago the little misses and fashion lovers of Italy dressed their hair in the same manner. I must say, however, that I believe the energy generally displayed by Americans in getting "a leetle" ahead of anybody or anything else, has leaked out in this, the hair-dressing line—for the medallion on this old tomb represents the *Volterra* maiden as first herself, then her hair. In those times, it being generally supposed that to have a head of hair the growth depended first on the creation of the women; but in these times, matters have swung completely around. The belief is beginning to gain ground that the body is only an offshoot of the chignon. Well, where tombs are created, there must lie the remains of the "once-living," and where there are bones there must be teeth. Wouldn't you like to see a set of teeth, Doctor, that did itself honor almost 3,000 years ago? I don't know where I had contracted the idea, but I had always supposed that folks, about that period, were generally larger in height and stature than people now-a-days; and to supply the demand of those big stomachs I had always imagined that larger teeth were carried around in bigger mouths. But, no sir! there it lies, a dainty little incisor, as perfect as when its owner "laid him down to sleep;" and among this rubbish, though rich rubbish for me, I hunted about for diseased members, and I was rewarded by gathering a great many; some decayed even with the process, others slightly. In a word, they were in those times as miserable, troublesome, worrying necessities to the livelihood of man as they are to-day. From the appearance of some of the maxillaries, with their quota of teeth, the owners must have died from pains of tooth ache. Periostitis, necrosis, exposed pulps, everything in exactly the same manner as the different diseases leave their impress at the present time.

I wondered at what a price "Taft's Operative Dentistry"

would have sold, or "Harris' Principles," &c., &c.; but there is one thing in which that little article referred to is wrong, and that is, the not ascribing to the ancients the science of filling teeth. Operative Dentistry may have been at a very low ebb, but I know of several gentlemen living at the present time, that would be willing to invest large sums to be able to learn the secret of the old white cement, that certain mummies possess and show in their teeth. It may have been the secret of one man alone; but, be that as it may, whenever we, with all our paraphernalia of Dental war, our big factories for gold foil, and the many articles used in saving teeth, can restore to its original shape, with its original color and a perfect imitation of an original enamel, a natural tooth, so that the place of its decay and disease is wholly obliterated, and the parts thereof perfectly restored, a preparation that resists the acids of the mouth, and changeless in color, then only can we hope that the apex of operative Dentistry is at last reached, and that we, with all our big notions, are as good at our trade as our grey-haired predecessors of some thousands of years standing.

And speaking of these things, permit me to mention a very interesting fact, and ask you for a solution: Almost every child that is born in Naples and stays there until he has shed his first teeth, for the second assortment of masticators has *red* teeth, in some instances *black*, *dentine and all* of these colors, and often the same tooth having the mixture. Whenever the first teeth are so, the second set is generally clear and white; but whenever, on the contrary, the milk teeth are like the stock in which we mortals in our babyhood generally invest, the second "edition" are on the mosaic style as already intimated. Let some wise-headed brother think about this. Naples contains 400,000 souls, and so there are too many proofs of the above if some of the profession should be inclined to doubt this.

